# What factors could influence physicians' management of women of childbearing age with chronic inflammatory disease? A systematic review of behavioural determinants of clinical inertia

**DOI:** 10.1186/s12913-019-4693-x

**Published:** 2019-11-21

**Authors:** Catherine Nelson-Piercy, Ivo Vlaev, Katie Harris, Rebecca Fischer-Betz

**Affiliations:** 1 Guy’s & St Thomas’ Foundation Trust and Imperial College Healthcare Trust, London, England; 2 Warwick Business School, University of Warwick, Coventry, England; 3 Ogilvy Health, Alphabeta Building, London, England; 4 Policlinic of Rheumatology and Hiller Research Unit Rheumatology, Medical Faculty, Heinrich-Heine-University, Moorenstr. 5, 40225 Düsseldorf, Germany

**Keywords:** Clinical inertia, Heuristics, Pregnancy, Chronic inflammatory disease, Behavioural science

## Abstract

**Background:**

Pregnancy represents a complex challenge to clinicians treating women with chronic inflammatory disease. Many clinicians face a situation of heightened sensitivity to the potential risks and uncertainties associated with the effect of pharmacological treatment on pregnancy outcomes. This may create an environment vulnerable to clinical inertia, whereby behavioural factors such as cognitive heuristics and biases, and other factors such as attitudes to risk and emotion can contribute. This systematic review was undertaken to assess if clinical inertia has been investigated/identified in this setting and took a behavioural science approach to identify and understand the potential determinants of clinical inertia in this treatment setting.

**Methods:**

A systematic literature search was conducted to identify publications which investigated or described clinical inertia or its determinants (e.g. heuristics, biases etc.). Results were coded for thematic analysis using two inter-related behavioural models: the COM-B model and the Theoretical Domains Framework.

**Results:**

Whilst studies investigating or describing clinical inertia in this treatment setting were not identified, the behavioural analysis revealed a number of barriers to the pharmacological management of women of fertile age affected by chronic inflammatory disease. Factors which may be influencing clinician’s behaviour were identified in all domains of the COM-B model. The primary factors identified were a lack of knowledge of treatment guidelines and fears concerning the safety of medications for mother and fetus. Lack of experience of treating pregnant patients was also identified as a contributing factor to undertreatment.

**Conclusion:**

Using a behavioural approach, it was possible to identify potential factors which may be negatively influencing clinician’s behaviour in this treatment setting, although specific research was limited.

## Background

Chronic inflammatory disease (CID), including chronic rheumatic diseases (CRDs) such as rheumatoid arthritis (RA), axial spondyloarthritis (axSpA), ankylosing spondylitis (AS), psoriatic arthritis (PsA), as well as other chronic conditions such as inflammatory bowel disease (IBD; Crohn’s disease and ulcerative colitis) and psoriasis (PsO), commonly affects patients during their reproductive years [1–5]. For women considering pregnancy and their treating clinician(s), there arise a number of concerns and uncertainties regarding the impact of active disease and continuing medication on conception, pregnancy outcomes and breastfeeding. This may create an environment vulnerable to ‘clinical inertia’.

Clinical inertia has typically been described in health conditions which take a ‘treat-to-target’ approach, such as Type 2 diabetes, hypertension and dyslipidaemia [6–8]. It was initially defined by Phillips et al. in 2001 as “failure of health care providers to initiate or intensify therapy when indicated” [9], but it has since been suggested that it be expanded to encompass other behaviours, such as prescription of preventative therapies and management of risk factors and complications [10]. In the setting of multiple sclerosis, an immune-mediated neurological disease with a pattern of flare and remission, clinical inertia has been defined as “lack of treatment initiation or escalation when there is evidence of disease activity” [11]. As a consequence of clinical inertia, patients can experience suboptimal management of their condition which carries increased risk of subsequent adverse health outcomes.

Treat-to-target has been established as a guiding principle for the treatment of patients with RA [12] and this approach has been shown to convey better outcomes compared with routine care [13]. Nevertheless, it seems that although some elements of treat-to-target are widely used, full implementation appears to remain uncommon [14].

Clinical inertia has been associated with behavioural factors such as mental heuristics and biases, attitudes to risk and emotion [15]. Humans use cognitive shortcuts (heuristics) to make a large number of decisions efficiently and effectively on a daily basis; however, when used inappropriately, these heuristics become biases, leading to suboptimal outcomes [16]. The involvement of heuristics and biases in medical decision making have been characterised by two systematic reviews [17, 18]. It appears that the use of heuristics in inappropriate contexts increases when the decision being made is associated with a degree of uncertainty or risk [18]. Clinical inertia also appears to be more prevalent when the treatment decision in question involves uncertainty or risk in terms of the effect on the patient’s outcome.

It was hypothesised that in the management of women of childbearing age with CID, clinical inertia could encompass not only a failure to initiate or escalate treatment, but also inappropriately discontinuing treatment (when it is required for disease control) due to uncertainty surrounding the risk of using of pharmacological agents during conception, pregnancy and breastfeeding. Therefore, clinical inertia and associated heuristics and biases may impact clinical decision making in the management of women of childbearing age with CID, leading to suboptimal disease management and patient outcomes. The objectives of this systematic review were to address the following research questions using a behavioural science approach: Has clinical inertia been described as affecting physicians' management of women of childbearing age with CID? What behavioural factors have been described as driving clinical inertia in this setting, such as the emotional context and physicians' attitudes to risk? Does the literature indicate that heuristics and biases are influencing clinical decision making in this setting?

Due to the qualitative nature of this literature review, barriers to the pharmacological management of women with CID identified were coded for thematic analysis using two inter-related behavioural models; the COM-B (Capability, Opportunity, Motivation, Behaviour) model and the Theoretical Domain Framework (TDF). The COM-B model proposes that human behaviour occurs due to the interaction between three necessary conditions: capability, which is the psychological and physical ability to enact the behaviour; opportunity, which can be both the physical and social environment that enables the behaviour; and motivation to perform the behaviour in preference to competing behaviours, which can be reflective (conscious, effortful, deliberative) and automatic (habits, emotional responses, impulses) [19]. The TDF has also been used extensively to understand behaviour across a wide range of healthcare and clinical settings. It contains 14 domains, which can be mapped against the COM-B model to gain a more ‘granular’ understanding of the determinants of the behaviour [20].

## Methods

### Literature search and paper selection

The literature search was carried out using the PubMed NCBI database in March 2018 (a search of the Cochrane database yielded no additional relevant results and will not be discussed further; a non-systematic search using Google Scholar was also performed to ensure no publications specifically investigating clinical inertia in this setting had been missed by using the PubMed database). Before conducting the search, all authors agreed on the major search terms and that if a search specific to CRD (RA, PsA, axSpA and AS) produced a low yield of results, then the search terms would be extended to include other CIDs (specifically Crohn’s disease, ulcerative colitis and psoriasis). For each stage of pregnancy (pre, during, and post-), 22 separate searches were conducted to identify records relevant to the theme of clinical inertia and cognitive biases in the therapeutic areas listed above (CRD and CID). A total of 66 different search strings were interrogated which identified 1466 records (Additional file 1: *Figure S1a and S1b details the search strings*). No date exclusions were applied.

Using the basic record information provided by PubMed as initial search output (title, author, journal, language), the following exclusion criteria were applied: publications not in English, in vitro or in vivo studies not in human subjects, or duplicate records of previous search strings. 569 publications were excluded. The abstracts of the remaining 897 publications were reviewed (KH) and categorised according to Table 1. A second researcher (NG) reviewed the categorisation and any discrepancies were discussed between the two parties (KH and NG) and resolved, resulting in 29 publications being assigned to category A and therefore selected for comprehensive analysis [1, 21–30, 31–34, 35, 36–48]. A flow chart of the selection process is presented in Additional file 1: Figure S2. A summary table of the 29 publications selected for comprehensive analysis is provided in Additional file 1: Table S1.

**Table 1:** Criteria for selection of papers for comprehensive analysis

Category	Description
A	Potential publication for full analysis. Abstract suggests that the full text may contain something relevant or of interest related to cognitive heuristics/biases, clinical inertia, or clinical decision making in the context of treating pregnant patients with CIDs
B	Publication is related to CRD/CID, pregnancy and treatment, but the abstract does not suggest that it contains any relevant information about heuristics/biases, clinical inertia or clinical decision making in the context of treatment pregnant patients with CIDs
C	Publication is about CRD/CID and pregnancy, but is unrelated to treatment, and is therefore unlikely to contain any relevant information for the purposes of this search
D	Publication is off-topic (wrong therapy area) and is therefore highly unlikely to contain any relevant information for this literature search

### Application of the COM-B model and TDF

The 29 publications were reviewed (by KH) for either a direct mention of a heuristic or bias or a barrier to management which could indicate clinical inertia. Quotations relevant to these themes were recorded from 17 publications [1, 21–30, 31–34, 35, 36]. Only quotations which reflected the views and opinions of clinicians were selected. Each quotation was then assigned a domain from the COM-B model of behaviour and a domain from the TDF (by KH) which most accurately reflected the type of behaviour being described (COM-B domains and the associated domains of the TDF, as well as the definitions and component constructs of the TDF domains can be found in Additional file 1: Table S2 and S3, respectively).

## Results

***“The desire to start a family adds additional complexity to management decisions preconception, during pregnancy and following delivery given the lack of safety data and potential teratogenicity of available therapies”*** [21]***.***

(For all selected quotations please refer to Additional file 1: Tables S4–S7)

**Fig. 1:**
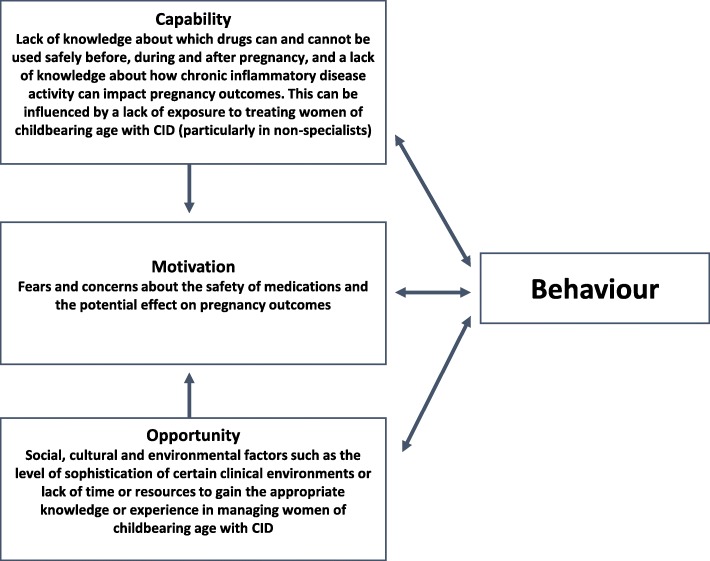
Application of the COM-B model (adapted from [19]) to the overarching findings of the literature search. Detailed findings of the literature search were categorised into capability, opportunity and motivation domains

No papers were identified that specifically investigated and thus confirmed the presence of clinical inertia in women of childbearing age with CID. However, an overarching opinion in many of the papers reviewed (and appeared to be true across both rheumatology and gastroenterology specialties) was that women of childbearing age with CID are considered complex and challenging cases to manage by clinicians [1, 21–26]. From this literature search it was possible to identify numerous factors that may be influencing optimal decision making in the management of CID patients and contributing to this label of ‘challenging’. By applying the COM-B model, it was possible to map these factors to all three domains – capability, opportunity and motivation – allowing identification of barriers and drivers that may be influencing clinician behaviour, and how these may contribute to heuristics and biases, and ultimately clinical inertia. Figure 1 summarises the overarching findings of the literature search as mapped onto the COM-B model.

### Capability domain

***“Deciding on an appropriate medication use during pregnancy is a challenge as there is a paucity of data regarding the safety of medications during pregnancy. This lack of data is primarily due to the ethical and logistical constraints of evaluating the safety of drugs during pregnancy”*** [27].

The dominant TDF domains that could be mapped to ‘capability’ were ‘knowledge’ and ‘skills’, both of which represent psychological capabilities. The knowledge gaps in clinicians treating pregnant patients could be due to the lack of robust and compelling evidence about the use of medications in pregnancy from well-designed, prospective, randomised controlled trials. This is mainly due to the ethical and logistical concerns of conducting clinical trials on medications in pregnant patients and the exclusion of pregnant women from many clinical trials. According to one paper analysed, much of the drug safety information available to clinicians is based on classification systems, voluntary reports or uncontrolled retrospective observational studies, which are often based on animal studies that use non-clinical doses of drugs [27].

### TDF domain: knowledge – psychological capability

***“Clinicians involved in the care of pregnant patients with inflammatory bowel disease (IBD) may not be aware of best practice guidelines, resulting in suboptimal patient care”*** [28]***.***

Knowledge of the current guidelines is important to optimise maternal and fetal outcomes [28]. In the knowledge domain, low awareness of clinical practice guidelines and general uncertainty about which drugs are considered safe to use in pregnancy was a topic explored in a number of publications [1, 28–30]. Clinicians may not be aware of clinical practice guidelines, and may also not be aware of the need to implement them due to a lack of knowledge of the effect of pregnancy on disease activity and vice versa, leading to suboptimal outcomes for patients [28, 29].

A number of publications that surveyed clinicians across a range of specialities revealed inadequate and variable knowledge regarding which drugs are considered compatible with pregnancy, particularly in non-specialists. In one publication, nearly half of GPs (44%) and obstetricians/gynaecologists (46%) believed that administering thiopurines (which are considered ‘low risk’ in pregnancy and breastfeeding in IBD guidelines [49]) would cause ‘serious harm’ to the baby [1]. Another publication reported that gastroenterology specialists were significantly more likely to advise patients to continue their IBD regimen (biologic agents and thiopurines) during pregnancy than non-specialists; biologic agents: 86% vs 46%; *P* < 0.0001 and thiopurines: 69% vs 15%; *P* < 0.0001) [28]. This same publication reported that almost 40% of clinicians were not aware of the need to delay administration of live vaccines to newborns after exposure to anti-TNF agents in utero [28].

This inadequate knowledge is concerning as unnecessary discontinuation of a therapy could put patients at risk of relapse and potential adverse pregnancy outcomes [1]. One publication concerning IBD in pregnancy concluded that only gastroenterologists were shown to have a ‘reliably high level of knowledge’, and that patients may be provided with inconsistent information about the use of medications during pregnancy depending on which type of physician they first encounter, which may heighten patient anxiety and fear [1].

### TDF domain: skills (competence, practice) – psychological capability

Adherence to guidelines appears to increase in clinicians who see a greater proportion of patients of childbearing age, and therefore have obtained the necessary knowledge and skills through experience to have confidence in their treatment decisions [28]. Competence, gained through practice, may therefore be an influencing factor in the management of women with CIDs. Conversely, a lack of competence may be a contributing factor to clinical inertia. Two publications analysed in this study made the association between the number of patients managed per year being predictive of a clinician’s clinical practice [28, 31]. One publication reported that clinicians who had treated more than 20 patients with IBD in the previous year were significantly more likely than those who had treated 0–4 patients to correctly manage a patient who was taking thiopurines or biologics and wanted to become pregnant (continue thiopurines, 54% vs 17%; *P* = 0.0014 and continue biologics, 79% vs 46%; *P* = 0.0074) [28].

### Motivation domain

***“The safety of medical therapy during pregnancy and lactation is a major concern for both pregnant women and their partners as well as for clinicians”*** [32]***.***

The dominating theme in the motivation domain appeared to be fears or concerns about the safety of medications for patients who are pregnant, breastfeeding or planning to become pregnant, which could be associated with a lack of knowledge regarding which therapies can be used in pregnancy, as outlined above. These fears and concerns would undoubtedly impact clinical decision making and potentially be a contributing factor to clinical inertia.

### TDF domain: social and professional role and identity – reflective motivation

One of the publications analysed reported patients’ opinions about their treating clinicians during their pregnancy [33]. Although the focus of this review was clinicians’ opinions, some insight could be gained from this. It was reported that the majority of patients see their rheumatologist as their primary information source and believed that their rheumatologist was capable of helping with treatment decision-making during their pregnancy. As such, patients put great trust in their rheumatologist regarding treatment decisions and pregnancy [33]. Whilst not alluded to in the publication itself, this could potentially be a source of further pressure on clinicians who may feel personally responsible for pregnancy outcomes and thus may anticipate feeling regret about their treatment decisions. This could translate into a more cautious approach and thus clinical inertia.

### TDF domain: emotion (fear, anxiety) – automatic motivation

***“Fear of medication effect on the fetus often prompts a clinician and/or patient to discontinue all medications”*** [34]***.***

Closely associated with the reflective ‘anticipation of regret’ domain is the automatic domain of emotion, in this case, fear and anxiety. In general, people prefer to make an error by omission (omission bias), that is, the outcome will be the consequence of an action not taken, rather than commission, where the outcome is related to actions actively taken [7]. Clinicians may fear that their pharmacological management of a patient’s condition during pregnancy, which they are responsible for, may lead to adverse outcomes for both mother and child, which may lead to suboptimal treatment decisions or discontinuation of all treatment when not clinically indicated. A lack of knowledge about the use of medications in pregnancy, as described above as part of the ‘Capability’ domain, may contribute to this fear and subsequent omission bias. Indeed, the COM-B model indicates that capability can have a direct influence on automatic motivation.

A number of publications reported asking clinicians about their comfort levels in treating pregnant patients. This is an interesting insight into clinicians’ emotional state when treating these patients. ‘Discomfort’ suggests a level of unease beyond simply a lack of knowledge, which is likely to influence risk-aversive heuristic behaviours. One publication reported that 79% of general practitioners and obstetricians/gynaecologists either felt uncomfortable or very uncomfortable in initiating IBD medication prior to or during pregnancy [1]. Another of these publications reported that physician comfort levels also influenced continuing pharmacological management of pregnant patients; clinicians who reported feeling comfortable with treating patients with IBD were significantly more likely than those who reported feeling uncomfortable to continue thiopurines (50% vs 19%, *P* = 0.0015) and continue biologics (75% vs 48%, *P* = 0.011) [28].

In terms of determining the source of clinicians’ fear and concerns in treating pregnant patients, one publication suggested that fears may be misplaced due to commonly used medications for autoimmune diseases having their pregnancy category determined from studies based on higher doses for a different indication. For example, thiopurines carried a pregnancy category D rating in response to the original submission for their use in high doses to treat leukaemia [34]. The FDA pregnancy categories have been updated because the five-letter system (A, B, C, D and X) was confusing, overly simplistic and did not reflect the available information about a drug [50]. In 2015, labelling changes were implemented, including information on pregnancy, lactation and information about contraception, fertility and the need for pregnancy testing [51]. Whether this change had any effect on clinicians’ prescribing behaviour was beyond the remit of this literature search, although it is possible that the biases that clinicians hold in their attitude towards certain medications in pregnancy may be hard to shift.

### Opportunity domain

As discussed previously as part of the capability domain, the influence of the sophistication of certain clinical environments (such as GP practices vs specialist units) and the subsequent yearly exposure to pregnant patients with CIDs has been identified as a factor which can influence clinicians' management of pregnant patients [28, 31]. This further reflects on how different domains of the COM-B can interact, that is, the opportunity to perform the behaviour in turn can increase capability. Additional factors related to opportunity that could contribute to clinical inertia were also identified from the literature search.

### TDF domain: environmental context and resources (resources, person and environment interaction)

One publication reported that whilst almost all GPs would consult specialists and obstetricians in managing IBD patients before conception, most felt unsupported or unsure as to how to contact a tertiary IBD service [1]; therefore they believe that the resources are not readily available to them. The time required for clinicians to physically research and educate themselves about the use of the various medications before, during and after pregnancy may also contribute to clinical inertia. One publication (which was not specifically describing this in relation to pregnancy) described the package inserts of biologic medicines to be ‘long and cumbersome’ and suggested that clinicians may shy away from their use due to the intricacies of each medication and the increasing number of them [35].

### TDF domain: social influences

***“This clinical practice guideline can be easy to disseminate and implement although it can be difficult to apply for several reasons within the context of our social and cultural environment”*** [29]***.***

One publication made the statement above regarding social and cultural influences affecting the dissemination of treatment guidelines [29]; however, the authors did not expand on what these may be. This could potentially be investigated further in cross-sectional behaviour studies.

## Discussion

***“Of paramount concern are questions about the effect of the disease on a woman’s ability to conceive and carry the pregnancy safely to term, as well as the effect of the disease and its therapies on the health of the fetus”*** [25]*.*

The fear of a medication’s effect on both the mother and fetus may encourage clinicians to inappropriately discontinue medication or fail to optimise treatment. In the case of IBD and CRD, uncontrolled disease activity is known to increase time to pregnancy and potentially negatively affect pregnancy outcomes [2, 3, 5, 34, 52–54]. However, how well this is understood or recognised by clinicians in the clinical management of these patients is unclear. Pregnancy therefore represents a somewhat unique situation to clinicians, who may not understand the risk associated with active disease, and therefore are not conditioned to think about the potential risk of discontinuing treatment in pregnancy. This also raises the question of whether discontinuation of therapy against guideline recommendations can be incorporated into the definition of clinical inertia, or whether this represents a distinct phenomenon.

The COM-B model and the TDF utilised in this study represented a systematic and comprehensive behavioural framework by which to assess both the individual and environmental influences on clinicians’ behaviour in this complex and under-reported reality. This allowed for a behavioural diagnosis of the potential drivers of clinical inertia. One of the key insights gained was around the low awareness of treatment guidelines for pregnant patients with CID. For some physicians, the lack of exposure to these patients means that they are unmotivated to invest the time to upskill themselves in the guidelines. In many hospital settings, a lack of active dissemination of guidelines places the responsibility on the clinicians to educate themselves. Even if a clinician does have sufficient knowledge of guideline recommendations, a lack of ability to explain risk to patients and/or a lack of experience doing this will impact the shared decision-making process. A feeling of a lack of support or unawareness of who to consult about guidelines may further impact their implementation. Social and cultural influences were also proposed to be factors affecting the dissemination and implementation of guidelines; however, the specifics of these were not reported by the authors and may warrant further exploration [29]. Since the studies investigated in this literature search predate the latest published guidelines of use of medications during pregnancy [49, 55], it would be of interest to investigate how the factors identified in this search reflects the current situation.

Fear about the consequences of teratogenicity is a strong affective component in the management of women of childbearing age, which likely stems from the experience with thalidomide and the more recent banning in the UK of valproate for women of childbearing age who are not enrolled in a pregnancy prevention programme [56]. The fear primarily sits with causing harm to the mother or baby, or both, however there also exist concerns about legal consequences and the high complexity of documentation in this context. Clinicians have to be very confident in their decisions in order to assure patients that a certain medication is compatible with pregnancy.

Another influence on the clinician’s pharmacological management of women with CID is the patient themselves and their concerns about the use of medications during pregnancy. This is in line with a shared decision-making approach recommended in many guidelines (for example the current EULAR guidelines for the treatment of RA [12]) where the patient’s perceptions of the risks associated with medication will also play a significant part in whether medication is discontinued or not. Outdated information about the safety of certain medications is a major issue in this context, represented by either incorrect information being provided to patients and clinicians from various sources, or based on historical and revised assumptions. An example of this is the inconsistent association between orofacial clefts and maternal use of corticosteroids despite this being disproven in numerous studies [57, 58]. Even with the appropriate knowledge and the motivation to prescribe a treatment, if clinicians fail to convince the patient that a certain medication is the optimal choice for them, then the opportunity to treat appropriately does not exist. Therefore, patient fears and concerns are likely to also impact clinical decision making.

The findings of this literature search suggest that clinicians managing women of childbearing age with CID could benefit from being made aware of clinical inertia as a concept, with interventions designed to educate and advise clinicians on how to recognise and overcome it. Clinicians could benefit from training on how to effectively communicate risk to patients – including the risk of active disease on their pregnancy along with the risks and benefits of the different treatment options available. Materials designed to aid this discussion (such as visuals aids) and that help patients clarify their individual preferences within the shared decision-making consultation could also be of benefit.

There is an increase in interest in the literature [7, 59–61] as to why clinical guidelines in general are not adequately adopted [59]. However, given the level of perceived risk in managing pregnant patients, it is somewhat surprising that more research has not been conducted in how this affects clinical decision making. Given the results of this literature search, it would be interesting to conduct a cross-sectional behavioural study of clinicians to determine the extent to which heuristics and biases and other factors identified in the literature review influence guideline adoption and clinical decision making in the pharmacological management of women of childbearing age with CRD. This may also provide the opportunity to develop and validate tools with which to measure or grade heuristics and biases in this context.

The use of PubMed as the main search database, and the lack of inclusion of other databases such as Embase, represents a limitation of the current study. A further limitation of this study is that the risk of bias was not formally assessed in the papers selected for comprehensive analysis. This was due to the qualitative nature of the study, and the lack of randomised controlled trials investigating this specific topic which could have made a formal assessment of bias possible.

## Conclusion

In conclusion, this literature search did not specifically identify any studies describing clinical inertia or heuristics/biases in the management of women of childbearing age with CID, nor did it elucidate if clinical inertia affects pregnancy outcomes – this in itself represents an unmet need in the literature. However, this search has identified that there are a number of factors that are likely to be influencing clinicians’ behaviour in this context, and therefore, it is likely that heuristics and biases are impacting the clinical decision-making process. These factors represent a starting-point for subsequent research investigating the specific attitudes and behaviours of clinicians in the management of women of childbearing age with CID.

## Additional file

**Additional file 1: Figure S1a** (top)**.** Search strings for the chronic rheumatic disease pre-, during- and post-pregnancy searches. **Figure S1b** (bottom): Search strings for the chronic inflammatory disease pre-, during- and post-pregnancy searches. **Figure S2a** (top): Analysis flow chart for the CRD search. **Figure S2b** (bottom). Analysis flow chart for the CID search. **Table S1.** Summary of publications selected for comprehensive analysis. **Table S2.** COM-B and associated domains of the TDF [20]. **Table S3.** TDF domains with definitions and component constructs [20]. **Table S4.** Quotations from publications on the topic of pregnancy being a complex or challenging condition to treat. **Table S5.** Quotations from publications in the capability domain. **Table S6.** Quotations from publications in the motivation domain. **Table S7.** Quotations from publications in the opportunity domain.

## Data Availability

All data generated or analysed during this study are included in this published article and its Additional file 1.

## Abbreviations

AS: Ankylosing spondylitis
axSpA: Axial spondyloarthritis
CID: Chronic inflammatory disease
COM-B: Capability opportunity motivation- behaviour
CRD: Chronic rheumatic disease
EULAR: European League Against Rheumatism
FDA: US Food and Drug Administration
GP: General practitioner
IBD: Inflammatory bowel disease
PsA: Psoriatic arthritis
PsO: Psoriasis
RA: Rheumatoid arthritis
TDF: Theoretical domains framework
TNF: Tumour necrosis factor
