# Supplementary material for: What factors could influence physicians' management of women of childbearing age with chronic inflammatory disease? A systematic review of behavioural determinants of clinical inertia
Source: BMC Health Serv Res. 2019 Nov 21;19:863. doi: 10.1186/s12913-019-4693-x (PMC6868709; doi:10.1186/s12913-019-4693-x)
Supplement: Supplementary file 1 — Additional file 1: Figure S1a (top). Search strings for the chronic rheumatic disease pre-, during- and post-pregnancy searches. Figure S1b (bottom): Search strings for the chronic inflammatory disease pre-, during- and post-pregnancy searches. Figure S2a (top): Analysis flow chart for the CRD search. Figure S2b (bottom). Analysis flow chart for the CID search. Table S1. Summary of publications selected for comprehensive analysis. Table S2. COM-B and associated domains of the TDF [20]. Table S3. TDF domains with definitions and component constructs [20]. Table S4. Quotations from publications on the topic of pregnancy being a complex or challenging condition to treat. Table S5. Quotations from publications in the capability domain. Table S6. Quotations from publications in the motivation domain. Table S7. Quotations from publications in the opportunity domain. [file 12913_2019_4693_MOESM1_ESM.docx]

**SUPPLEMENTARY MATERIALS**

**Supplementary Figure 1a (top): Search strings for the chronic rheumatic disease pre-, during- and post-pregnancy searches. Figure 1b (bottom): Search strings for the chronic inflammatory disease pre-, during- and post-pregnancy searches.**


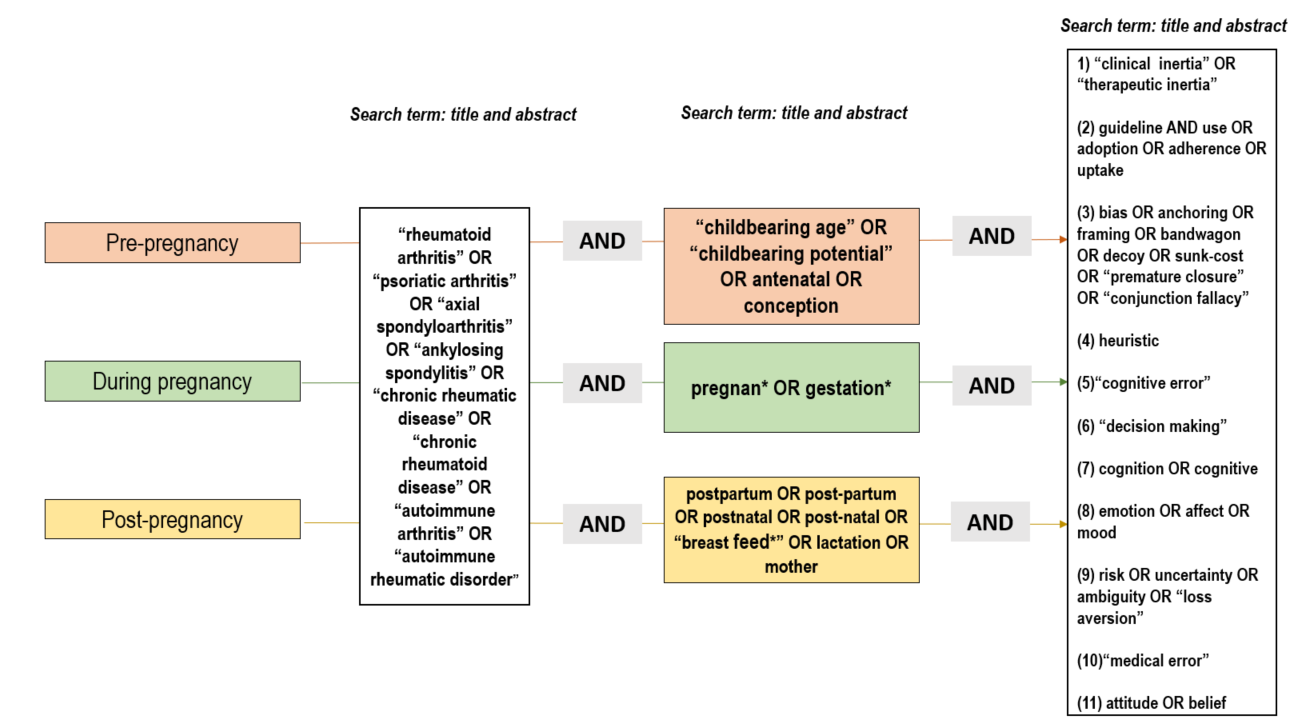


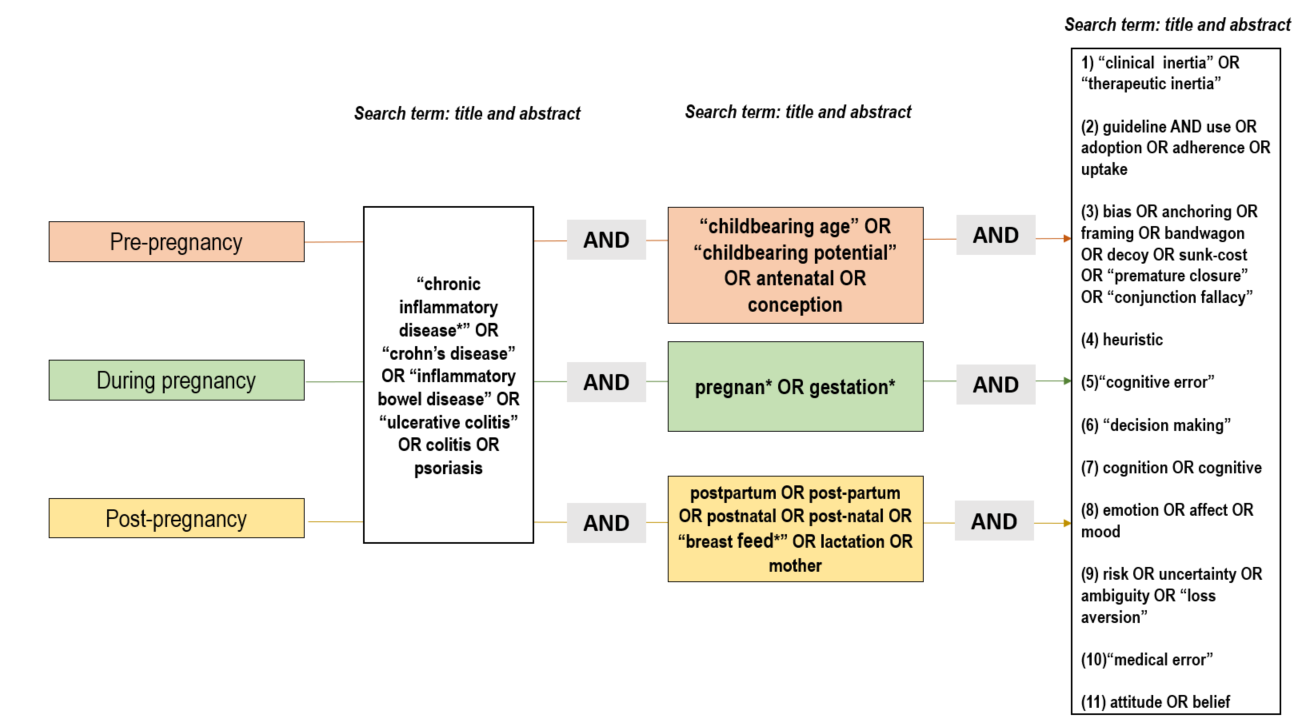


**Supplementary Figure 2a (top): Analysis flow chart for the CRD search. Figure 2b (bottom): Analysis flow chart for the CID search**


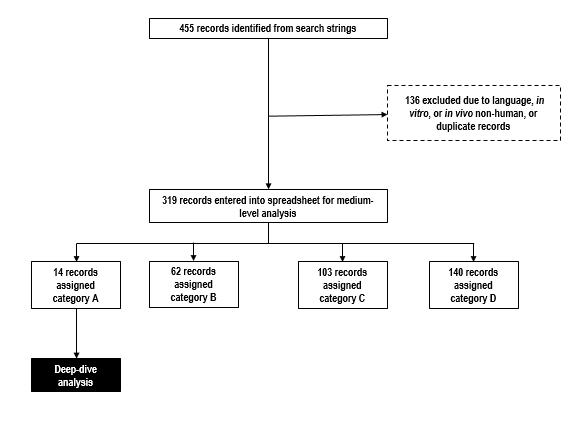


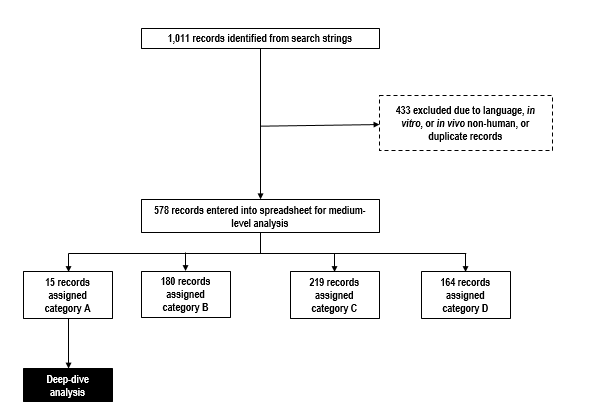


**Supplementary Table 1. Summary of publications selected for comprehensive analysis.**

| **Author** | **Year of pub.** | **Search theme** | **Therapy area** | **Study type** | **Study groups/population of interest** | **Sample sizes** | **Country** | **Objectives/research question** | **Key findings** |
| --- | --- | --- | --- | --- | --- | --- | --- | --- | --- |
| Ackerman IN, et al. | 2015 | Decision making | RA | Interviews & focus groups | Women with RA who were pregnant in the last 5 years, currently pregnant or planning pregnancy | N=27 | Australia | To determine the need for (and preferred mode/s of delivery of) information regarding pregnant, post-natal care and early parenting among women with RA. | Lack of information about medication safety, access to physical/emotional support services and practical strategies for coping with daily challenges related to parenting were the most prominent of the six key themes identified. Rheumatologists were the primary source for information regarding treatment decisions while arthritis consumer organisations were perceived as critical ‘resource hubs’. There was strong preference for information delivered electronically, especially among rural participants. |
| Barbhaiya M & Bermas BL | 2013 | Risk and uncertainty | Rheum | Review | Women of childbearing age with RA and SLE | n/a | n/a | To discuss the effect of pregnancy on patients with the most common rheumatic diseases, the effect of the diseases on the pregnancy itself, and the management of patients during pregnancy. | Pregnancy is associated with an increased risk in adverse maternal and fetal outcomes. Patient’s disease should be in good control and ideally in remission prior to conception. It is important to understand what medication options are acceptable for patients to take during pregnancy in order to develop a treatment plan. Close collaboration between the obstetrician and rheumatologist during pregnancy is essential. |
| Beaulieu DB & Kane S | 2011 | Emotion | IBD | Review | Women of childbearing age with IBD | n/a | n/a | To discuss the evidence for the important questions that female patients have in regard to family planning and IBD (how does IBD affect the outcomes of pregnancy and how does pregnancy affect the disease course). To provide recommendations based on clinical experience of the authors. | Potential risks are involved with pregnancy in patients with IBD. Conversations with the patient should occur before conception. Continued monitoring and aggressive control of the disease prior to conception and throughout combined with effective shared decision-making is essential to achieve optimal outcomes for mother and baby. |
| Beaulieu DB & Kane S | 2011 | Emotion | IBD | Review | Women of childbearing age with IBD | n/a | n/a | To review the available data regarding fertility, conception, medication, safety, pregnancy, delivery and breastfeeding for women with IBD. | Risks are involved in pregnancy in patients with IBD but successful outcomes are possible. Remission before conception is the goal and medication choices should be discussed with the patient prior to pregnancy. IBD medications during pregnancy are low risk (except MTX and thalidomide). Shared decision-making between patients, obstetricians and the gastroenterologist is important. |
| Belilos E & Carsons S | 1998 | Emotion/ affect/ mood | Fibromyalgia, RA, Sjögren’s syndrome, SLE, APS | Review | Women of childbearing age with rheumatic diseases | n/a | n/a | To review common rheumatic diseases that most frequently occur in women. | Many rheumatological autoimmune and connective tissue diseases occur in young and middle-aged women. In this group of patients, many women are of childbearing potential and special considerations for pregnancy arise. |
| Biedermann L, et al. | 2012 | Risk | IBD | Review | Women of childbearing age with IBD | n/a | Europe | To review recent developments on the topic of pregnancy and breastfeeding in IBD consistent with the ECCO guidelines. | A large majority of patients conceive and carry a pregnancy under safe conditions. The precondition to this is patient awareness and helping them to wait for and attain a remission before considering pregnancy, and to deploy all means to keep them in remission during the gestational period. The benefit of continuing medical therapy in IBD during pregnancy outweighs the potential risks in the vast majority of instances. |
| Borchers AT, et al. | 2010 | Risk and uncertainty | SLE, MS, RA, T1D | Review | Women of childbearing age with autoimmune diseases | n/a | n/a | Discuss the implications of autoimmune diseases and pregnancy with regards to safety of the mother and foetus, disease course, pregnancy outcomes and therapeutic management. Discuss the female predominance of autoimmune diseases and if it is related to microchimerism. | Epidemiological studies have contributed to advances in treatment for women with autoimmune diseases who wish to become pregnant. Combined with careful, multidisciplinary management in the preconception and pregnancy period, the outlook in essentially all autoimmune diseases has improved considerably. |
| Chakravarty FF, et al. | 2003 | Risk and uncertainty | RA | Survey | Rheumatologists | N=175 | US | To describe the practice of rheumatologists when prescribing DMARD MTX, LF, ET and IN to women of childbearing age with RA and the pregnancy outcomes of patients who become pregnant whilst taking these medications. | Rheumatologists agree on the risk of teratogenicity with MTX and LF and the need for birth control. No consensus about ET and IN but birth control still discussed. Confirmed risk of congenital malformations with in utero exposure to MTX. |
| Danion P, et al. | 2018 | Risk | IBD | Survey | IBD patients | N=364 | France | To develop an updated self-questionnaire to assess patients’ level of knowledge of IBD. | Created an updated questionnaire that enables health care providers to more fully assess patient knowledge about IBD. Reported a lack of knowledge about IBD patients that justifies improving and rethinking methods of informing patients to increase their health and well-being. The areas of knowledge least mastered were vaccination, IBD-related cancers, treatments and pregnancy. |
| Gupta R, et al. | 2013 | Risk | IBD | Article | Women of childbearing age with dermatological conditions | n/a | n/a | To introduce the medicolegal risks involved in prescribing dermatological medications to a pregnant patient and how to mitigate those risks. | Medication use in pregnant patients for the treatment of a skin condition poses a significant legal risk to the dermatologist. Pharmacological treatment is warranted if the potential benefits of the treatment outweigh the risks. Dermatologists should be aware of the risks and adequately discuss and document the patient’s understanding of the potential side effects. |
| Habal FM & Huang VW. | 2012 | Decision-making | IBD | Review | Women of childbearing age with IBD | n/a | n/a | To develop an evidence-based decision-making algorithm to help guide physicians through the management of pregnancy in the IBD patient. | A number of important concepts were identified for the management of women with IBD: preconception counselling on various issues, better pregnancy outcomes are in patients in remission, drugs to treat IBD are generally safe during pregnancy (excluding MTX and CsA) and lactation (excluding CsA), biologics can be continued for up to 30 weeks gestation, management should be multidisciplinary and involve the patient. |
| Kashkooli SB, et al. | 2015 | Risk | IBD | Survey | GPs, OB/GYNs, GEs | N=337 | Australia | To objectively measure IBD-specific pregnancy-related knowledge of GPs and OB/GYNs in comparison to GEs using the validated CCPKnow questionnaire. | GPs had significantly lower knowledge than GEs for the composite CCPKnow and almost all domains. GEs were the only group to attain a median CCPKnow score in the top category. More than 70% of GPs and OB/GYNs expressed discomfort with initiation of IBD medications around conception/pregnancy. GPs (43.6%) and OB/GYNs (45.7%) perceived thiopurine use to be unsafe during pregnancy and to cause serious harm to the baby. |
| Keeling SO & Oswald AE. | 2009 | Emotion | RA, SLE | Review | Women of childbearing age with rheumatic diseases | n/a | n/a | To review the general state of pregnancy and how it is affected by prototypical rheumatic diseases including RA and SLE. To present the most commonly used DMARD drugs and immunosuppressants and explain the difference between FDA category and clinical practice among rheumatologists. Provide general recommendations on how to manage rheumatic disease in pregnancy. | Autoimmune diseases commonly affect women of childbearing age. Clinical experience has shown a select groups of medications, if monitored properly, can be maintained during pregnancy. Some of these would not be considered based on FDA categories alone. Preconception planning is essential. |
| Krause ML et al. | 2014 | Decision making | RA | Review | Women of childbearing age with RA | n/a | n/a | To provide evidence-based recommendations for the use of DMARDs and biologics to guide rheumatologists in the care of pregnant and lactating women. | To optimise the changes of a healthy pregnancy, the following should be taken into account: the most suitable therapy according to pregnancy desire, education regarding appropriate contraception, appropriate timing of pregnancy when RA is stable, adequate follow-up and treatment post-partum. Early and thorough dialogue with the patient is very important. |
| Mahadevan U & Matro R. | 2015 | Risk | IBD | Review | Women of childbearing age with IBD | n/a | n/a | To outline important consideration for OBs caring for women with IBD before and during pregnancy and in the post-partum period. | Women with IBD require a multidisciplinary approach to maximise their chances of a successful, healthy pregnancy. Most medications used in the management of IBD can be continued during pregnancy and lactation. A multidisciplinary treatment plan established before conception can reduce the chance of discontinuation of effective medications and lead to more successful outcomes. |
| Makol A, et al. | 2011 | Risk and uncertainty | RA | Review | Women of childbearing age with RA | n/a | n/a | To provide an overview of the RA treatment issues pre-conception, during pregnancy and in the post-partum period with respect to breastfeeding. To provide guidelines for drugs that may be used relatively safely for RA management in pregnant women. | Short- and long-term pregnancy desires should be considered when selecting immunomodulator therapy for women with RA. Education about the risks of certain treatments is key to avoid unplanned pregnancies. A successful pregnancy is best achieved with disease quiescent pre-pregnancy, safe medication during pregnancy and close follow-up post-partum. Disease flares can be managed with corticosteroids. |
| Marder W | 2016 | Emotion | RA | Review | Women of childbearing age with autoimmune connective tissue diseases | n/a | n/a | To provide an overview of pregnancy as a risk factor for onset of autoimmune disease, considerations related to the course of pregnancy in several autoimmune connective tissue diseases, and disease management and medication issues before and during pregnancy and the postpartum period. | Active maternal disease during pregnancy is associated with adverse pregnancy outcomes. Maternal and fetal health can be optimised when conception is planned during times of inactive disease and through maintaining treatment regimens compatible with pregnancy. |
| Meade T, et al. | 2013 | Bias | RA | Qualitative study and thematic analysis | Women of childbearing age with RA | N=14 | Australia | To examine women’s experiences of negotiating their family decisions in the context of RA. | Three key themes were identified which affected women’s motherhood decision-making process and experiences: capacity, uncertainty and acceptance. Women face challenges when contemplating motherhood and there is a need for understanding and support from HCPs and the provision of materials to help women make informed choices. |
| Mottet C, et al. | 2009 | Decision-making | CD | Treatment recommend-ations based on an expert panel | Special situations in CD | n/a | n/a | To develop appropriateness criteria for upper gastro-intestinal, extra-intestinal manifestations and drug safety during conception, pregnancy and breastfeeding in patients with Crohn's disease, to assist the physician in clinical decision making. | Azathioprine/6-MP, prednisone and mesalamine are considered safe throughout pregnancy. Infliximab is considered safe at the time of conception of during the first trimester but uncertain during the second and third trimester. A slightly more cautious approach is recommended concerning azathioprine/6-MP and sulfasalazine use in breastfeeding. |
| Ng SW & Mahadevan U | 2013 | Guidelines | IBD | Review | Women of childbearing age with IBD | n/a | n/a | To review the nuances of IBD management in women who are considering pregnancy, attempting to conceive or already pregnant. | HCPs should balance the risks of medication to the foetus with the risks of stopping therapy and the impact of subsequent disease activity to both mother and the fetus. |
| Ng SW & Mahadevan U | 2014 | Risk | IBD | Review | Women of childbearing age with IBD | n/a | n/a | Outline the approach to the treatment of women with IBD with respect to preconception counselling and management during pregnancy and the postpartum period. | Pregnant women with IBD are at increased risk of adverse outcomes and should be observed as high-risk patients (even in remission). Ideally quiescent or stable disease should be achieved before conception to reduce the risk of adverse outcomes. Most IBD medications can be safely continued during pregnancy. Preconception counselling and a medical therapy plan agreed with the patient is important, and a multidisciplinary approach towards management should be taken. |
| Ortleb M & Levitt J. | 2012 | Risk | Derm | Article | Dermatology patients eligible for biologics | n/a | n/a | To create checklists and discuss considerations behind various counselling and monitoring issues for biologic treatment. | Consensus is unavailable as to the extent of counselling or monitoring. Checklists were developed for a number of commonly used biologics to use in an office setting and allow for a standardised approach to counselling and monitoring. |
| Østensen M | 2014 | Risk | RA | Review | Women of childbearing age with RA | n.a | n/a | To encourage discussion of reproductive issues in all patients of fertile age in order to prevent unplanned and ill-timed pregnancies in patients with RA. | Preconception counselling and risk assessment is important in women with RA, with a particular focus on preventing unplanned pregnancy by information on contraception. Antibody status and all medications need to be reviewed before pregnancy. Maintaining low disease activity before and during pregnancy is crucial for good outcomes. |
| Østensen M | 2017 | Risk and uncertainty | Rheum | Review | Women of childbearing age with rheumatic disease | n/a | n/a | Provide an overview of preconception counselling. | A well-functioning and open route of communication between HCPs engaged in the care of an individual patient is important when counselling a patient about the timing of therapy during therapy. Pregnancy outcomes for mother and child are likely to be better when patients are well informed and actively engaged in decision-making before conception, during pregnancy and in the post-partum period. |
| Peyrin-Biroulet L, et al. | 2011 | Risk | CD | Survey | GEs | N=175 | Worldwide | To perform a worldwide survey for evaluating the extent to which GEs who are experts in the field of IBD are utilising thiopurines during pregnancy and in the post-operative setting in Crohn’s disease. | Almost 9 of 10 physicians continue azathioprine throughout pregnancy. |
| Rhodes R, et al. | 2014 | Risk | IBD | Survey | HCPs (FM, IM, OB-GYN, GE) | N=116 | US | To assess practitioners in different specialties who are frequently involved in the care of pregnant patients with IBD and determine their awareness regarding the safety of using IBD medication in this patient population. | Practitioners involved in the care of pregnant patients may be unaware of how to manage IBD drugs safely during pregnancy. This is especially true of physicians who see relatively few patients with IBD. |
| Saavedra Salinas MÁ. et al. | 2015 | Guidelines | SLE, RA, APS | Practice guideline | Women of childbearing age with SLE, RA, APS | n/a | Mexico | To provide recommendations regarding prenatal care, treatment, and a more effective monitoring of pregnancy in women with SLE, RA and APS. | An expert panel developed treatment algorithms and recommendations related to maternal and fetal care and the use of antirheumatic drugs during pregnancy and lactation. |
| Thompson AE & Bashook PG. | 2010 | Risk and uncertainty | RA | Survey | Rheumatologists | N=170 | Canada | To determine what key information patients must know about MTX and the key reasons they should call their doctor while they are taking MTX. | In patients taking MTX, regular blood testing, once-weekly dosing, risk of infection, pregnancy and lactation information, alcohol limitation, potential lung toxicity, and drug interactions were thought to be important. Patients should call their doctor if they became pregnant, developed symptoms suggestive of lung toxicity or an infection, severe mouth sores or were concerned about any side effects. |
| Vader JP, et al. | 2006 | Decision-making | CD | Treatment recommend-ations based on an expert panel | CD patients | n/a | Europe | To develop appropriateness criteria for different aspects of therapy for CD. | Specific pregnancy considerations were as follows: MTX is contraindicated. Sulfasalazine, mesalazine and prednisone were considered safe, and infliximab and budesonide were rated uncertain at any stage of pregnancy. AZA/6-MP is considered safe in later pregnancy (second and third trimester) but uncertain in early pregnancy or during nursing. Antibiotics were considered unsafe in early pregnancy and uncertain in the later stages of pregnancy or during nursing. |

Abbreviations: 6-MP: 6-mercaptopurine; APS: antiphospholipid syndrome; AZA: azathioprine; CCPKnow: Crohn’s and Colitis Pregnancy Knowledge questionnaire; CD: Crohn’s disease; CsA: ciclosporin; Derm: dermatology; DMARD: disease-modifying antirheumatic drug; ECCO: European Crohn’s and Colitis Organisation; ET: etanercept; FDA: US Food and Drug Administration; FM: family medicine; GE: gastroenterologist; GP: general practitioner; GYN: gynaecologist; HCP: Healthcare professional; IBD: inflammatory bowel disease; IM: internal medicine; IN: infliximab; LF: leflunomide; MS: multiple sclerosis; MTX: methotrexate; OB: obstetrician; RA: rheumatoid arthritis; SLE: systemic lupus erythematosus; T1D: type 1 diabetes

**Supplementary Table 2. COM-B and associated domains of the TDF (Atkins L, et al. 2017)**

| **COM-B** | **Theoretical domain** |
| --- | --- |
| Physical capability | Physical skills |
| Psychological capability | Knowledge |
|  | Memory, attention and decision processes |
|  | Behavioural regulation |
| Reflective motivation | Professional/social role and identity |
|  | Beliefs about capabilities |
|  | Optimism |
|  | Beliefs about consequences |
|  | Intentions |
|  | Goals |
| Automatic motivation | Reinforcement |
|  | Emotion |
| Physical opportunity | Environment context and resources |
| Social opportunity | Social influences |

**Supplementary Table 3. TDF domains with definitions and component constructs (Atkins L, et al. 2017)**

| **Domain** | **Constructs** |
| --- | --- |
| **1. Knowledge** (an awareness of the existence of something | Knowledge (including knowledge of condition/scientific rationale, procedural knowledge, knowledge of task environment |
| **2. Skills** (an ability or proficiency acquired through practice) | Skills, skills development, competence’ ability, interpersonal skills, practice, skill assessment |
| **3. Social/professional role and identity** (a coherent set of behaviours and displayed personal qualities of an individual in a social or work setting) | Professional identity, professional role, social identity, identity, professional boundaries, professional confidence, group identity, leadership, organisational commitment |
| **4. Beliefs about capabilities** (acceptance of the truth, reality or validity about an ability, talent or facility that a person can put to constructive use) | Self-confidence, perceived competence, self-efficacy, perceived behavioural control, beliefs, self-esteem, empowerment, professional confidence |
| **5. Optimism** (the confidence that things will happen for the best or that desired goals will be attained) | Optimism, pessimism, unrealistic optimism, identity |
| **6. Beliefs about** consequences (acceptance of the truth, reality, or validity about outcomes of a behaviour in a given situation) | Beliefs, outcome expectancies, characteristics of outcome expectancies, anticipated regret, consequents |
| **7. Reinforcement** (increase the probability of a response by arranging a dependent relationship, or contingency, between the response and a given stimulus) | Rewards (proximal/distal, valued/not valued, probable/improbable), incentives, punishment, consequents, reinforcement, contingencies, sanctions |
| **8. Intentions** (a conscious decision to perform a behaviour or resolve to act in a certain way) | Stability of intentions, stages of change model, transtheoretical model and stages of change |
| **9. Goals** (mental representations of outcomes or end states that an individual wants to achieve) | Goals (distal/proximal), goal priority, goal/target setting, goals (autonomous/controlled), action planning, implementation intention |
| **10. Memory, attention and decision processes** (the ability to retain information, focus selectively on aspects of the environment and choose between two or more alternatives) | Memory, attention, attention control, decision making, cognitive overload/tiredness |
| **11. Environmental context and** **resources** (any circumstance of a person’s situation or environment that discourages or encourages the development of skills and abilities, independence, social competence and adaptive behaviour) | Environmental stressors, resources/material resources, organisations culture/climate, salient events/ critical incidents, person x environment interaction, barriers and facilitators |
| **12. Social influences** (those interpersonal processes that can cause individuals to change their thoughts, feelings, or behaviours) | Social pressure, social norms, group conformity, social comparisons, group norms, social support, power, intergroup conflict, alienation, group identity, modelling |
| **13. Emotion** (a complex reaction pattern, involving experiential, behavioural, and physiological elements, by which the individual attempts to deal with a personally significant matter or event) | Fear, anxiety, affect, stress, depression, positive/negative affect, burn-out |
| **14. Behavioural regulation** (anything aimed at managing or changing objectively observed or measured actions) | Self-monitoring, breaking habit, action planning |

**Supplementary Table 4. Quotations from publications on the topic of pregnancy being a complex or challenging condition to treat**

| **Quotation** | **Therapy area** | **Author, year** |
| --- | --- | --- |
| ***“Managing pregnant patients with rheumatic disease can be very challenging”*** | CRD | Keeling *et al,* 2009 |
| ***“Pregnancy can pose a challenge to the clinician caring for women with rheumatoid arthritis”*** | RA | Makol *et al,* 2011 |
| ***“The RA context, therefore, compounds the complexity of the motherhood decision and presents a challenge for women with RA and the health professionals who manage their care”*** | RA | Meade *et al*, 2013 |
| ***“The desire to start a family adds additional complexity to management decisions preconception, during pregnancy and following delivery given the lack of safety data and potential teratogenicity of available therapies”*** | RA | Krause *et al,* 2014 |
| ***“The interactions between pregnancy and IBD are complex, presenting challenges both for patients and health care professionals (HCPs) who treat and advise them”*** | IBD | Kashkooli *et al,* 2015 |
| ***“Management of IBD in pregnancy poses special challenges to the healthcare provider”*** | IBD | Ng *et al,* 2013 |
| ***“Growing experience with IBD in pregnancy suggests that most women will have good outcomes; however, concerns and uncertainty remain for both the patient and the clinician”*** | IBD | Ng *et al,* 2014 |

**Supplementary Table 5. Quotations from publications in the capability domain**

| **Quotation** | **Therapy Area** | **Author, year** | **TDF domain** |
| --- | --- | --- | --- |
| ***“The limited knowledge of clinicians who are not rheumatologists as regards the effect of pregnancy on the autoimmune disease and vice versa may also be a limitation for its [guideline] whole implementation”*** | CRD | Saavedra Salinas *et al*, 2015 | Knowledge |
| ***“Clinicians involved in the care of pregnant patients with IBD may not be aware of best practice guidelines, resulting in suboptimal patient care”*** | IBD | Rhodes *et al,* 2014 | Knowledge |
| ***“In contrast to current guidelines, 43.6% of GPs believed administering thiopurine during pregnancy might cause serious harm to the baby or was ‘considered unsafe’, while only 17% thought that the therapy could continue. Only 11% answered that infliximab and adalimumab were regarded as ‘probably safe’ to use during pregnancy”*** | IBD | Kashkooli *et al*, 2015 | Knowledge |
| ***“An almost equal number [of clinicians] indicated that they were uncertain about the safety of pregnancy with the use of these medications (49.7% ET [etanercept] and 45.4% IN [infliximab])”*** | RA | Chakravarty *et al*, 2003 | Knowledge |
| ***“Compared with GI clinicians, FM+IM clinicians were less likely to correctly identify infliximab***  ***as a pregnancy category B drug (67% vs 30%; P=0.0005)”*** | IBD | Rhodes *et al,* 2014 | Knowledge |
| ***"Among OB/GYNs, despite the encouraging global scores, nearly half (46%) thought that the use of azathioprine (AZA) or 6-mercaptopurine (MP) during pregnancy would cause serious harm to the baby or was ‘considered unsafe’. Only 32% thought that AZA/MP could continue, and only 32% thought that infliximab and adalimumab were regarded as ‘probably safe’ to use during pregnancy”*** | IBD | Kashkooli *et al,* 2015 | Knowledge |
| ***“GI specialists were more likely to advise patients to continue their IBD regimen (biologic agents and thiopurines) during pregnancy than non-GI (IM, FM, and Ob/Gyn) clinicians (biologic agents: 86% vs 46%; P<0.0001 and thiopurines: 69% vs 15%; P<0.0001)”*** | IBD | Rhodes *et al,* 2014 | Knowledge |
| ***“Among all respondents, 38% were unaware of the need to delay administration of live vaccines to infants exposed to anti-tumour necrosis factor agents in utero”*** | IBD | Rhodes *et al,* 2014 | Knowledge |
| ***“Both GPs and OB/GYNs demonstrated poor understanding of the safety and use of thiopurines, infliximab and adalimumab, which may lead to inappropriate patient management, unnecessary and dangerous cessation of maintenance medications and/or failure to initiate medications when needed. Not only may this heighten patient anxiety and fears around pregnancy and reproduction, it may also jeopardise IBD disease control and pregnancy outcomes”*** | IBD | Kashkooli *et al,* 2015 | Knowledge |
| ***“It is clearly shown that only GEs [gastroenterologists] have a reliably high level of knowledge, making it uncertain what information IBD patients will get depending on which OB/GYN or GP they encounter”*** | IBD | Kashkooli *et al,* 2015 | Knowledge |
| ***“Practitioners who followed more than 100 patients per year were more likely to maintain treatment with azathioprine throughout the pregnancy, whereas practitioners following less than 100 patients tend to never prescribe azathioprine during pregnancy”*** | Crohn’s disease | Peyrin-Biroulet *et al,* 2011 | Skills |
| ***“Clinicians managing a greater number of patients with IBD seemed to be more aware of current guidelines”*** | IBD | Rhodes *et al,* 2014 | Skills |
| ***“The number of patients followed per doctor was predictive of their clinical practice”*** | Crohn’s disease | Peyrin-Biroulet *et al,* 2011 | Skills |
| ***Clinicians who had treated more than 20 patients with IBD in the past year were more likely than clinicians who had treated 0 to 4 patients with IBD in the past year to correctly manage a patient with IBD who wanted to become pregnant in both scenarios (continue thiopurines, 54% vs 17%; P=0.0014 and continue biologics, 79% vs 46%; P=0.0074)*** | IBD | Rhodes *et al,* 2014 | Skills |

**Supplementary Table 6. Quotations from publications in the motivation domain**

| **Quotation** | **Therapy area** | **Author, year** | **TDF domain** |
| --- | --- | --- | --- |
| ***“The majority of telephone interviewees cited their rheumatologist as their primary information source, particularly in relation to medication decision-making. Participants spoke more about trusting their rheumatologist’s judgment rather than wanting to understand detailed information relating to RA medications”*** | RA | Ackerman *et al,* 2015 | Social and professional role and identity |
| ***“Our quantitative findings closely reflect the qualitative data, in that women strongly desired further information yet placed great trust in their treating rheumatologist, particularly regarding medication decisions”*** | RA | Ackerman *et al,* 2015 | Social and professional role and identity |
| ***“When a woman of childbearing age is diagnosed with RA, the rheumatologist is entrusted with multiple responsibilities”*** | CRD | Krause *et al,* 2014 | Social and professional role and identity |
| ***“Until recently, many medications were discontinued peripartum for fear of adverse pregnancy outcomes”*** | CRD | Keeling *et al,* 2009 | Emotion |
| ***“Despite the expressed confidence with IBD-specific pregnancy related issues overall, the majority of GPs (78.7%) felt either very uncomfortable or uncomfortable in starting IBD-related medication prior to or during pregnancy”*** | IBD | Kashkooli *et al,* 2015 | Emotion |
| ***“Clinicians who reported feeling comfortable treating patients with IBD were more likely than clinicians who rated themselves as uncomfortable treating patients with IBD to correctly manage a patient with IBD who wanted to become pregnant (continue thiopurines, 50% vs 19%; P=0.0015 and continue biologics, 75% vs 48%; P=0.011)”*** | IBD | Rhodes *et al,* 2014 | Emotion |
| ***“Comfort levels in treating IBD patients: V comfortable 11%, Comfortable 20%, Neutral 19%, Uncomfortable 32%, V uncomfortable 18%”*** | IBD | Rhodes *et al,* 2014 | Emotion |
| ***“The thiopurines, AZA and 6-MP, carry a pregnancy category D rating. This was in response to the original FDA submission for their use with high doses to treat leukaemia. Since then, low doses have been used for autoimmune diseases and become the standard of care without a change in the FDA label, leading to misplaced fears and concerns by patients, gastroenterologists, and obstetricians”*** | IBD | Beaulieu *et al,* 2011 | Emotion |
| ***“Fear of medication effect on the fetus often prompts a clinician and/or patient to discontinue all medications. Pregnancy data on outcomes and disease course are complicated by the cessation of drugs, but the risk of complications during pregnancy seems primarily related to disease activity and not medication effect”*** | IBD | Beaulieu *et al,* 2011 | Emotion |

**Supplementary Table 7. Quotations from publications in the opportunity domain**

| **Quotation** | **Therapy Area** | **Author, year** | **TDF domain** |
| --- | --- | --- | --- |
| ***“Almost all GPs would consult IBD specialists and obstetricians in managing IBD patients or their medication use before conception. However, most GPs felt either unsupported or unsure as to how to contact a tertiary IBD service”*** | IBD | Kashkooli *et al,* 2015 | Environmental context and resources |
| ***“As with all medications, the biologics come with their fair share of risks, which require pre-treatment screening and ongoing monitoring. The package inserts of these agents are often long and cumbersome. Unfortunately, because of the intricacies of each individual agent and the growing variety of agents, some clinicians shy away from their use”*** | Dermatology | Ortleb *et al,* 2012 | Environmental context and resources |
| ***“This clinical practice guideline can be easy to disseminate and implement although it can be difficult to apply for several reasons within the context of our social and cultural environment”*** | CRD | Saavedra Salinas *et al,* 2015 | Social influences |
